# Reproductive State Modulates Retinal Sensitivity to Light in Female Túngara Frogs

**DOI:** 10.3389/fnbeh.2019.00293

**Published:** 2020-01-21

**Authors:** Caitlin E. Leslie, Robert F. Rosencrans, Whitney Walkowski, William C. Gordon, Nicolas G. Bazan, Michael J. Ryan, Hamilton E. Farris

**Affiliations:** ^1^Department of Integrative Biology, The University of Texas at Austin, Austin, TX, United States; ^2^Neuroscience Center, Louisiana State University School of Medicine, New Orleans, LA, United States; ^3^Department of Cell Biology and Anatomy, Louisiana State University School of Medicine, New Orleans, LA, United States; ^4^Department of Ophthalmology, Louisiana State University School of Medicine, New Orleans, LA, United States; ^5^Smithsonian Tropical Research Institute, Balboa, Panama; ^6^Department of Otorhinolaryngology, Louisiana State University School of Medicine, New Orleans, LA, United States

**Keywords:** retina, visual sensitivity, hCG (human chorionic gonadotropin), hormone/reproduction/sexual, túngara frogs

## Abstract

Visual cues are often a vital part of animal communication and courtship. While a plethora of studies have focused on the role that hormones play in acoustic communication of anurans, relatively few have explored hormonal modulation of vision in these animals. Much of what we do know comes from behavioral studies, which show that a frog’s hormonal state can significantly affect both its visual behavior and mating decisions. However, to fully understand how frogs use visual cues to make these mating decisions, we must first understand how their visual system processes these cues, and how hormones affect these processes. To do this, we performed electroretinograms (ERGs) to measure retinal sensitivity of túngara frogs (*Physalaemus pustulosus*), a neotropical species whose mating behavior includes previously described visual cues. To determine the effect of hormonal state on visual sensitivity, ERGs were recorded under scotopic and photopic conditions in frogs that were either non-reproductive or hormone-treated with human chorionic gonadotropin (hCG) prior to testing. Additionally, measurements of optical anatomy determined how túngara frog eye and retina morphology related to physiological sensitivity. As expected, we found that both sexes display higher visual sensitivity under scotopic conditions compared to photopic conditions. However, hormone injections significantly increased retinal sensitivity of females under scotopic conditions. These results support the hypothesis that hormonal modulation of neural mechanisms, such as those mediating visually guided reproductive behavior in this species, include modulation of the receptor organ: the retina. Thus, our data serve as a starting point for elucidating the mechanism of hormonal modulation of visual sensitivity.

## Introduction

Visual cues, especially in the context of reproductive behavior, are important in many animal communication systems (for review, see Candolin, 2003), subjecting the visual system to strong selection. For example, ultraviolet plumage in birds (Andersson and Amundsen, 1997), light flash properties in fireflies (Branham and Greenfield, 1996; Vencl and Carlson, 1998), and caudal appendages in swordtails (Basolo, 1990) are all visual traits that are well known to affect mating behaviors and mate preferences. In order to modulate behavior, these visual traits must first be encoded by the retina and subjected to further central processing in the brain. Although endocrine mechanisms mediate reproductive behavior in general (Rhen and Crews, 2002), the extent to which they modulate the receptor organs during reproductive communication is largely unknown.

Compared to central neural modulation, there are relatively few data demonstrating direct hormonal modulation of sensory epithelia. For example, direct hormonal modulation has been shown in the inner ear of plainfin midshipman fish (Sisneros et al., 2004), the olfactory epithelium in tinfoil barbs (Cardwell et al., 1995), and electroreceptors in a weakly electric fish (Keller et al., 1986; Meyer et al., 1987). Fewer still are studies that investigate hormonal modulation of the retina. We are aware of only one recent study making such direct measurements, showing mouthbrooding African cichlid females have a higher expression of sex steroids in retinal tissue as well as heightened retinal sensitivity (Butler et al., 2019). That study notwithstanding, compelling evidence for this phenomenon in other systems is largely indirect or lacking measures of sensitivity change in the sensory organ itself. For example, histological approaches have revealed steroid hormone receptors in the retinas of humans (Ogueta et al., 1999), goldfish (Tchoudakova et al., 1999), western mosquitofish, sailfin mollies (Friesen et al., 2017), and three-spined sticklebacks (Hoffmann et al., 2012). With respect to behavior, female and male three-spined sticklebacks show increased spectral sensitivity (as measured by optomotor response) to red light (which is characteristic of courting male coloration) during the breeding season (Cronly-Dillon and Sharma, 1968; Boulcott and Braithwaite, 2007). Physiological studies (Shao et al., 2014) were consistent with these behavioral (optomotor) studies and specifically indicated that steroid hormones drive this retinal modulation. Additionally, retinal sensitivity in *Anolis* lizards shows a circadian rhythm that is dependent on an intact pineal gland, implicating modulation by melatonin (Shaw et al., 1993). Even when taken together, these studies still show that our understanding of how hormones modulate visual sensitivity is incomplete. Our goal is to address this gap by making direct electrophysiological measures of retinal sensitivity in a model anuran system.

Frogs are visual animals (Lettvin et al., 1959; Ewert, 1987); even nocturnal species see well at night (Rosencrans et al., 2018). In some species, visual cues, such as vocal sac or body coloration, mediate aspects of their mate choice (Summers et al., 1999; Gomez et al., 2009; Starnberger et al., 2014). The túngara frog (*Physalaemus pustulosus*; genus also referred to as *Engystomops*), in particular, is an excellent species in which to study the effects of hormones on vision. These frogs integrate visual cues with their acoustic communication (Rosenthal et al., 2004; Taylor et al., 2008, 2011b; Taylor and Ryan, 2013). Specifically, male mating calls consist of a frequency sweep or “whine,” followed by 0–7 harmonic bursts called “chucks.” Whereas the whine is necessary and sufficient to attract a female, chucks enhance the call’s attractiveness in choice tests (Rand and Ryan, 1981). During call production, the vocal sac inflates and deflates in concert with the call. Although anuran vocal sacs have evolved, in part, to facilitate air exchange during calling (Bucher et al., 1982; Pauly et al., 2006), the túngara frog vocal sac adds a visual cue that enhances the salience of the acoustic signal in mate searching females. When given a choice between an attractive conspecific call alone and that same call synchronized with a video playback of a calling male, females prefer the audiovisual stimulus to the call alone (Rosenthal et al., 2004). A robotic frog with an inflating vocal sac also increases the attractiveness of a conspecific call under specific conditions (Taylor et al., 2008, 2011a,b). Although an inflating vocal sac alone is neither necessary nor sufficient for a female to approach a potential mate (Taylor et al., 2011b), these behavioral studies show that what a female sees can affect her mating decisions, creating the opportunity for selection on the visual system to act differently in males and females.

The hormonal modulation of mate choice behavior in túngara frogs, including through hormonal manipulation in the lab, has been well documented. Much of that work has focused on auditory behavior and central auditory processing (Lynch et al., 2006; Chakraborty and Burmeister, 2009, 2015; Baugh and Ryan, 2017). But there is also evidence for a role of hormones in visual behavioral sensitivity, as light intensities that elicit optokinetic responses (OKR) in female túngara frogs vary by reproductive state, with reproductive females showing lower visual behavioral thresholds (Cummings et al., 2008). These behavioral data do not indicate the anatomical locations of this modulation, however. Thus, this study addresses what mechanism could underlie increased female sensitivity by determining whether the mechanisms include the retina.

The first step in this process is to determine the visual sensitivity of the túngara frog retina; in other words, to determine what a túngara frog is physiologically capable of seeing, much the same way previous studies have determined what this same frog is physiologically capable of hearing (Ryan et al., 1990; Wilczynski et al., 2001). Just as the long history of auditory communication studies began in the ear, our goal here is to add a new perspective on túngara visual communication by focusing on the first step in visual processing – the eye.

The second step is to examine the effects of manipulating reproductive state on the sensitivity of the túngara frog retina. This study used histological and electrophysiological techniques, respectively, to determine the relationship between optical and retinal sensitivity and whether this relationship changes under a reproductive hormonal state. Electroretinograms measured retinal sensitivity in awake male and female túngara frogs. Subjects were either non-reproductive or injected with human chorionic gonadotropin (hCG), which modulates frog CNS function and hormone production (Yang et al., 2007; Lynch and Wilczynski, 2008), and drives ovulation and mating behaviors in túngara. These data indicate hCG is sufficient to initiate a switch in reproductive state (Lynch et al., 2006; Chakraborty and Burmeister, 2009). Our data show that the physiological response of the retina to light, much like the previously established behavioral response, increases in hormonally modulated females only, consistent with the conclusion that hormonal modulation of the receptor organ is a component of endocrine control of mating behavior, potentially modulating the processing of visual mating signals. Furthermore, this endocrine mediated shift in retinal threshold enables females to use the predicted full extent of optical sensitivity created by the anatomy of their eye. This threshold shift introduces an underappreciated consideration to the relationship between optical and retinal sensitivity: the endocrine or reproductive state of the animal.

## Materials and Methods

All animal care, experimental and analysis methods are based on our previous work in frogs (Rosencrans et al., 2018).

### Research Animals

All experiments were approved by the Institutional Animal Care and Use Committees of the University of Texas at Austin; Louisiana State University Health Sciences Center, New Orleans; and the Smithsonian Tropical Research Institute. Study samples included lab-reared frogs from our colony at the University of Texas at Austin as well as wild-caught frogs from Panama. The subject species (*Physalaemus pustulosus*; túngara frogs) was chosen based on two primary criteria. First, they use vision during reproductive behavior, including the evaluation of sexual signals during nocturnal mate searching (Taylor et al., 2008, 2011a,b). Second, protocols are established for experimentally inducing reproductive behavior in the lab, including mate searching and egg laying (Lynch et al., 2006; Chakraborty and Burmeister, 2009). All frogs were housed either individually or in same-sex groups to prevent breeding behavior. The housing was an ‘a-seasonal’ environment: they were fed *ad libitum* and kept in a 12:12 light/dark cycle (300 cd/m^2^), with temperature (23.3°C) and humidity held constant (>60%). Thus, there were no cues of wet (reproductive) vs. dry season. When tissue collection was necessary, animals were euthanized using 150 mg/kg intramuscular ethyl 3-aminobenzoate (Tricaine methanesulfonate; MS-222; Sigma Aldrich) and then decapitated.

### Electroretinograms

Prior to ERG recordings, frogs were dark adapted for at least 12 h in a light-tight box. All subsequent preparation for recordings was conducted under dim red light (650 nm). Frogs were immobilized using an intramuscular injection of succinylcholine chloride (15 μg/g; Sigma-Aldrich; St. Louis, MO, United States), and atropine sulfate (1%) was applied to both eyes to maintain pupil dilation. Frogs were then placed on a damp towel under an Espion Ganzfeld Dome (Diagnosys LLC; Lowell, MA, United States) in a dark Faraday cage (0 lux; Extech HD450 photometer). Subdermal needle electrodes (GRASS Technologies or Harvard Apparatus) were inserted at the vertex of the skull and in the leg for indifferent and ground recordings, respectively. Silver-chloride electrodes were placed on the corneas of both eyes enabling simultaneous ERG recordings. The ERG from only one eye – that with the largest signal-to-noise ratio - was used for analysis. Experiments included both scotopic and photopic ERGs, enabling tests of sensitivity in primarily rod and cone dominated vision, respectively. Considering visual ecology, the separate tests allow for analysis of whether there are sexual and reproductive related differences in retinal sensitivity under nocturnal (scotopic) and diurnal (photopic) conditions. Both scotopic and photopic ERG procedures began with a 6-min adaptation time to allow for recovery from dim red light exposure. For the scotopic procedure, the adaptation time consisted of darkness and was followed by a series of 1 ms light flashes at 21 different, increasing light intensities (0–2,000 cd s/m^2^) with four sequential flashes at each intensity. There was no illumination between flashes. For the photopic procedure, the adaptation time and the time between flashes consisted of a constant background illumination (1.45 log cd/m^2^) that is within the range of daytime light intensities of the túngara frog’s natural habitat (Jaeger and Hailman, 1981). The adaptation time was followed by light flashes at 16 different, increasing light intensities (0–3,000 cd s/m^2^), again in sets of four at each given light intensity. Inter-flash intervals (5–120 s) and inter-step intervals (30–120 s) increased as light intensity increased to prevent retinal light adaptation. Recordings took place within ∼2 h before and ∼4 h after artificial (the frogs’) sunset. With respect to sex, light condition, and hormone treatment, ERGs were run in random order. Thus, no time of day or season is correlated with the ERG responses and their metrics.

### Hormone Treatment

ERG recordings were conducted in two experimental categories of frogs: control or experimentally induced reproductive state. To induce a reproductive state, immediately prior to dark adaptation, frogs were given a subcutaneous injection of human chorionic gonadotropin (500 IU; Sigma) dissolved in (50 μl) saline solution (in mM): 126 NaCl, 0.5 KCl, 2.8 CaCl_2_, 2.2 MgCl_2_, and 10 NaHEPES, pH 7.4 (274 mOsm). After injection, each frog was placed in a plastic tank with a frog of the opposite sex and provided with damp moss and access to water. The frogs were then placed in dark adaption and the ERG was run using the recording procedure described above. Lynch et al. (2006) established this hormone injection protocol in *P. pustulosus*, showing that females exhibit oviposition, increased estrogen, and phonotaxis 20–24 h after injection. In our study, females likewise responded to the injections by producing eggs (67%). Additionally, previous work using this protocol showed that there is no change in reproductive state pre versus post injection of saline alone. Furthermore, saline fails to elicit female phonotaxis, which is consistently exhibited by amplexed or hCG injected individuals (Lynch et al., 2006; Chakraborty and Burmeister, 2009). Thus, in the present study the control group remained in endogenous non-reproductive condition, received no injection, were housed alone, and followed the ERG recording procedure above.

### Electroretinogram Analysis

While ERG recordings were taken from both eyes, only the recording from the eye with the highest signal-to-noise ratio was used to determine threshold, saturation, and slope of the V-Log(I) function, which shows the relative b-wave amplitude as a function of stimulus luminance. Based on our earlier study in frogs (Rosencrans et al., 2018), the b-wave amplitude was defined as the difference between the average voltage over 20 ms before the light flash and the maximum voltage between 50 and 400 ms after the flash. The initial step for each procedure consisted of four recordings (sweeps) with no light flash, enabling correction for DC potential in recordings. The response to each intensity step consisted of the average of four flashes of the same intensity. Note that in some cases one of the four responses was removed from the average if either the electrode became uncoupled from the cornea or extraneous noise (e.g., heartbeat) prevented determination of a b-wave peak in the averaged trace. Because the absolute b-wave amplitude varied between individuals due to extracellular recording factors (e.g., electrode-cornea coupling and resistance), the intensity response function, or V-Log(I) curve, for each individual was normalized to that individual’s maximum b-wave amplitude. This resulted in a relative response curve with responses ranging from 0 to 1 (Miller and Dowling, 1970). Consistent with our previous work (Rosencrans et al., 2018), response threshold was defined as the light intensity eliciting a response 10% the amplitude of the maximum response. This light level was calculated after analyzing each individual V-Log(I) curve using a least-squares fit of the standard Boltzmann function:

(1) $$R\,e\,l\,a\,t\,i\,v\,e\,\text{b-wave}\,a\,m\,p\,l\,i\,t\,u\,d\,e=\frac{A_{1}-A_{2}}{1+e^{\frac{\left(f\,l\,a\,s\,h-f\,l\,a\,s\,h_{0}\right)}{\tau }}}+A_{2}$$

In this equation, A_1_ is the starting amplitude (0) and A_2_ is the ending amplitude (1); *flash* is the log intensity of each light flash; *flash*_0_ is the light intensity causing a 50% response; and τ is the slope of the function. We use this function to compare response thresholds across sexes and treatment groups in this study (Eguchi and Horikoshi, 1984).

### Optical Measurements

Optical sensitivity was calculated using the Land equation (Eq. 2), in which sensitivity (S) is the ratio of photons absorbed by a photoreceptor to those emitted within a steradian (sr) of solid angle of an extended source (Warrant and Dacke, 2011).

(2) $$S={\left(\frac{\pi }{4}\right)}^{2}\,{\left(A\right)}^{2}\,{\left(\frac{d}{f}\right)}^{2}\,\left(\frac{k\,l}{2.3+k\,l}\right)$$

**FIGURE 1:**
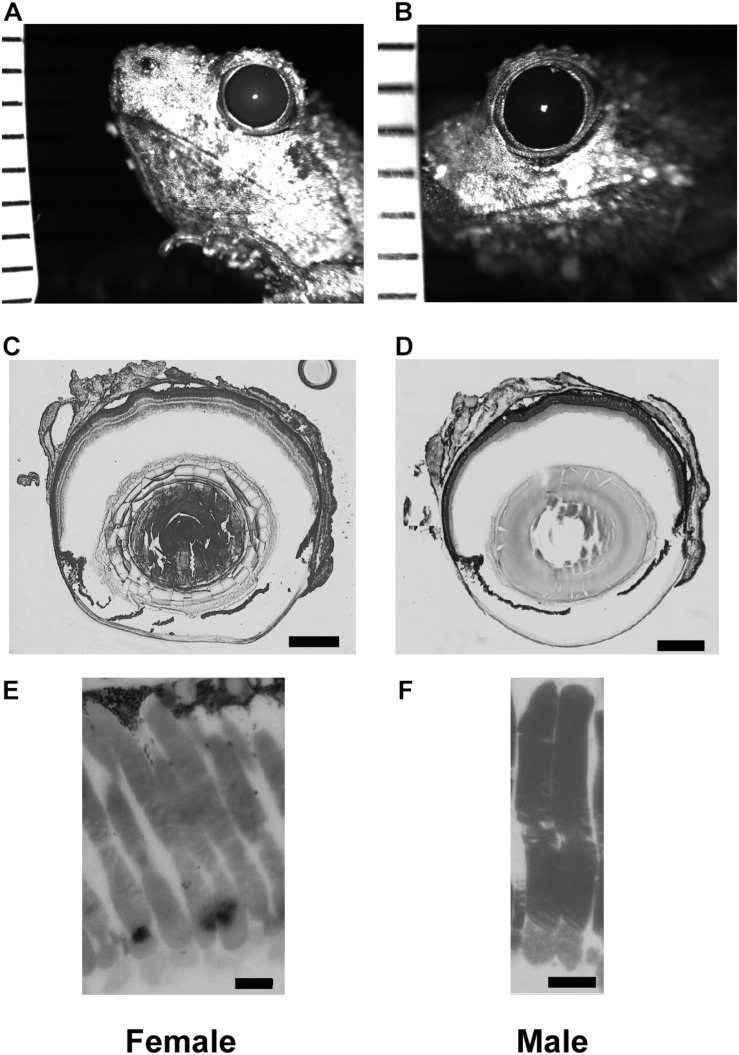
Tissue enabling optical measurements used in Land equation to calculate sensitivity for females (left column) and males (right column). **(A,B)** Example infrared photographs of dilated pupils used to measure aperture size. Tick marks are 1 mm to calibrate digital measurements. **(C,D)** Example flash frozen sections at largest lens diameter used to measure focal distance. Scale bar = 0.5 mm. **(E,F)** High magnification light microscopy of rod outer segments. Scale bar = 10 μm. Measurement comparisons are in Tables 1, 2 and Supplementary Figure S1.

Here, *A* is the aperture, *f* is the focal length, and *d* and *l* are the diameter and length of a rod outer segment, respectively (Land, 1981; Warrant and Nilsson, 1998; Land and Nilsson, 2002). *k* represents the absorption coefficient, or the proportion of photons absorbed per unit length of the photoreceptors (0.041 for frogs: Liebman, 1972; Hárosi and MacNichol, 1974; Warrant and Nilsson, 1998). The units for S are μm^2^ sr. To determine focal length measurements, eyes were extracted and flash-frozen in tissue media (OCT compound) with liquid nitrogen, and then sectioned at 20–60 μm. These sections were stained with toluidine blue, and the sections with the widest lens were used to measure focal length and lens width. The distance from the center of the lens to the junction between the photoreceptor outer segments and inner segments was measured under a light microscope with a calibrated reticule (Figure 1).

**TABLE 1:** Variation in ERGs and optical anatomy.

	*Female Untreated*		*Female hCG*		*Male Untreated*		*Male hCG*	
	Mean ± SD	(*n*)	Mean ± SD	(*n*)	Mean ± SD	(*n*)	Mean ± SD	(*n*)
**Scotopic ERG**								
Threshold	−2.90 ± 0.51	(10)	−4.22 ± 0.72	(7)	−3.17 ± 0.67	(6)	−3.26 ± 0.35	(5)
Saturation	2.97 ± 0.84	(10)	2.20 ± 1.28	(7)	2.54 ± 1.20	(6)	1.38 ± 0.26	(5)
Slope	1.34 ± 0.22	(10)	1.46 ± 0.38	(7)	1.30 ± 0.26	(6)	1.06 ± 0.08	(5)
**Photopic ERG**								
Threshold	−0.20 ± 0.22	(9)	−0.38 ± 0.17	(5)	−0.48 ± 0.40	(9)	−0.54 ± 0.34	(7)
Saturation	2.67 ± 0.34	(9)	2.20 ± 0.31	(5)	2.83 ± 0.47	(9)	2.68 ± 0.39	(7)
Slope	0.65 ± 0.12	(9)	0.59 ± 0.04	(5)	0.75 ± 0.12	(9)	0.73 ± 0.13	(7)
**Optical Anatomy**								
OS length (*l*) (mm)	69.40 ± 7.46	(30)			52.54 ± 6.65	(30)		
OS diameter (*d*) (mm)	7.13 ± 0.83	(30)			7.20 ± 1.08	(30)		
Focal length (*f*)(mm)	1.57 ± 0.10	(6)			1.57 ± 0.07	(6)		
Pupil (*A*)(mm)	1.98 ± 0.09	(6)			1.98 ± 0.10	(6)		
Sensitivity (*S*)(μm^2^ sr)	28.05 ± 5.26	(6)			24.80 ± 3.56	(6)		

*The left column shows the measures from the V-Log(I) curves and optical anatomy. Subsequent columns are the mean (±standard deviation) and sample size for each measure from the four treatment groups. hCG refers to subjects injected with human chorionic gonadotropin. Threshold and saturation are reported in Log cd s/m^2^ and are calculated from the Boltzmann fits to the V-Log(I) curves. OS refers to rod outer segment.*

**TABLE 2:** Statistical comparisons of mean ERG thresholds, ERG Slopes, and Optical anatomy.

			Test statistic
*Measurement*	*Comparison*		and *P*-values
**Thresholds**			
	Scotopic male	Photopic male	*P* < 0.0001^∗^
	Scotopic female	Photopic female	*P* < 0.0001^∗^
	Scotopic male	Scotopic female	*P* = 0.9437
	Photopic male	Photopic female	*P* = 0.9008
	hCG Scotopic female	Scotopic female	*P* < 0.0001^∗^
	hCG Scotopic male	Scotopic male	*P* = 1.000
	hCG Scotopic female	hCG Scotopic male	*P* = 0.0182^∗^
	hCG Photopic male	Photopic male	*P* = 1.000
	hCG Photopic female	Photopic female	*P* = 0.9963
**Slopes**			
	Scotopic male	Photopic male	*P* = 0.0001^∗^
	Scotopic female	Photopic female	*P* < 0.0001^∗^
	Scotopic male	Scotopic female	*P* = 1.000
	Photopic male	Photopic female	*P* = 0.9619
	hCG Scotopic female	Scotopic female	*P* = 0.9072
	hCG Scotopic male	Scotopic male	*P* = 0.4860
	hCG Scotopic female	hCG Scotopic male	*P* = 0.0235^∗^
	hCG Photopic male	Photopic male	*P* = 1.000
	hCG Photopic female	Photopic female	*P* = 0.9991
**Optical Anatomy**			
	Female vs. Male rod outer segment length (*l*)		*F* = 36.44, *P* = 0.0038^†^
	Female vs. Male rod outer segment diameter (*d*)		*F* = 2.6, *P* = 0.46
	Female vs. Male Optical Sensitivity (*S*)		*t* = 1.2, *P* = 0.25
	Female vs. Male focal distance (*f*)		*t* < 0.0001, *P* = 1.00
	Female vs. Male aperture diameter (*A*)		*t* = 0.066, *P* = 0.95

*ERG metrics are taken from the Boltzmann fits to the V-Log(I) curves. For ERG thresholds and slopes, the right column shows *P*-values for ANOVA *post hoc* testing, which were corrected for multiple comparisons (Tukey–Kramer procedure). Asterisks (^∗^) denote significant difference. ^†^Denotes differences that are significant using a nested ANOVA (outer segment measures are nested within individual and within sex). Mean optical sensitivity, focal distance, and aperture are compared using student’s *t*-tests. See Table 1 for values in all comparisons.*

Pupillary diameter was measured from infrared images (Figures 1A,B). Frogs were dark adapted for at least 2 h, after which 1% atropine sulfate (Sigma-Aldrich; St. Louis, MO, United States) was applied to their corneas. The pupils were then imaged (2007 Heidelberg Spectralis infrared camera, Heidelberg Engineering; Carlsbad, CA, United States) with a ruler in the same focal plane for calibration. Pupillary size was measured with software calipers (Heidelberg 6 software) and pupillary diameter calculated as an average of the rostral-caudal and dorsal-ventral axes lengths. The same eyes were used for pupillary diameter and focal length measurements (Figures 1C,D).

Rod dimensions were measured from 1 μm plastic sections (Figures 1E,F). All retinas were harvested at the same time, within 3 h of artificial sunset. Retinas were fixed overnight in Karnovsky’s fixative (2% glutaraldehyde, 2% formaldehyde, 0.1M sodium cacodylate buffer: Electron Microscopy Sciences; Hatfield, Pennsylvania) and then rinsed with buffer and post-fixed in cacodylate-buffered 1% osmium tetroxide (Electron Microscopy Sciences) for 1 h. They were then dehydrated in an ascending series of ethanol and acetone, infiltrated in 1:1 acetone to epoxy (Embed-812/araldite mixture), and polymerized in pureresin overnight at 56°C. The retinas were then sectioned at 1 μm and stained with 1% w/v aqueous toluidine blue and sodium borate. The measured rods were chosen from superior to inferior slices of the retina within 20 degrees of the optic nerve. Outer segments from 3 males and 3 females (30 cells from each sex) were measured using a light microscope with a calibrated reticule attachment. Although using thin slice plastic sections may limit analysis of some cells not sectioned at their longest or widest point, the method yields results not different from those using whole mounted retina and an adjustable focal plane (e.g., DIC microscopy; Rosencrans et al., 2018). Land optical sensitivity (Eq. 2) was calculated using the aperture and focal distance measures for 6 males and 6 females (within eye *A* and *f*). Because the equation uses only one value of *A*, *f*, *l*, and *d* per eye, the mean rod outer segment diameter and length was used for each eye’s calculation. This is consistent with previous methodology in frog models (Rosencrans et al., 2018).

### Statistical Analysis

ERG V-Log(I) curves, normalized to their maximum response amplitude, were analyzed with a least squared fit of the Boltzmann function. For each curve, this fit enabled calculation of the light intensities eliciting responses at 10% (threshold) and 90% (saturation) of the maximum amplitude, as well as calculation of the function’s slope. The effects of the three treatments (sex, background light, hCG; 2 × 2 × 2) were tested using a three-way ANOVA model (SAS software), which enabled subsequent comparison of the differences in the means of the V-Log(I) thresholds and slopes. Alpha values were corrected to account for multiple comparisons (Tukey–Kramer procedure). Male versus female mean optical sensitivity, aperture, and focal distance were assessed using Student’s *t*-tests. The effect of sex on rod outer segment dimensions, however, was analyzed using a nested ANOVA to accommodate sampling multiple rods from single retinas: rod measures were nested within individual and within sex. This enabled reporting of F statistics and *P*-values representing only the variance explained by sex.

## Results

### Scotopic and Photopic ERG Thresholds in Untreated Frogs

**FIGURE 2:**
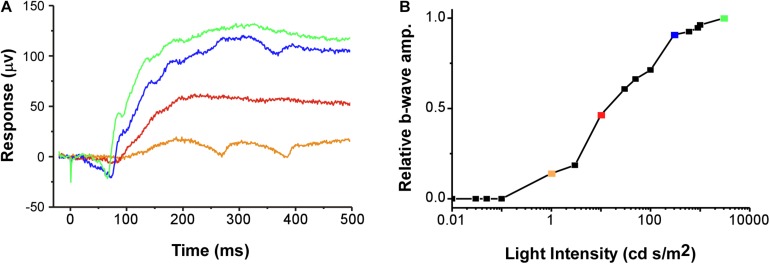
**(A)** Example of raw data for typical ERG waveforms (voltage trace) in response to a 1 ms duration flash of light starting at 0 ms (stimulus artifact occurs from 0 to 1 ms of recording). Example traces are from four of the 16 light steps (1 no-light and 15 light intensity steps). **(B)** The relative amplitude of the b-wave is plotted as a V-Log(I) curve. Colors of the example voltage traces (in **A**) correspond to the symbols on the plot, with each point denoting a different relative response (proportion of maximum) along the V-Log(I) curve for this recording. The b-wave amplitude was defined as the difference between the average voltage over 20 ms before the light flash and the maximum voltage between 50 and 400 ms after the flash (Rosencrans et al., 2018). Green symbol and trace are the maximum response (1.0). The example is from a photopic recording in an uninjected female.

**FIGURE 3:**
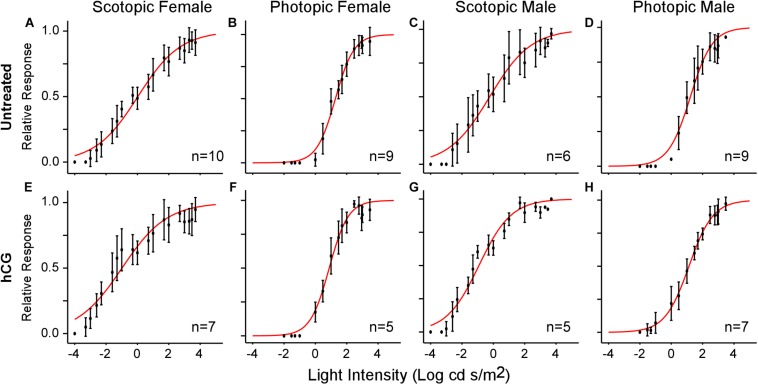
V-Log(I) curves showing mean ERG responses for each sex under scotopic and photopic conditions. Each point represents the mean (±SD) relative b-wave amplitude at each light intensity. Sample size (n) indicates the number of frogs used in each test. **(A–D)** V-Log(I) curves for untreated frogs. **(E–H)** V-Log(I) curves for hCG injected frogs. Red curves are the least-squares fit of the Boltzmann function to the entire population data. Note that Boltzmann function fits for each individual response were used to calculate mean threshold (i.e., light level eliciting 10% of maximum b-wave amplitude), slope, and dynamic range.

All ERGs conformed to the typical waveform (Figure 2A), consisting of a- and b-waves resulting from the responses of photoreceptors and bipolar cells, respectively (Pugh et al., 1998; Robson and Frishman, 1998). When the b-wave amplitudes (V) for each stimulus light intensity (I) are normalized to the maximum response amplitude for each individual, V-Log(I) curves exhibit sigmoidal change (Figure 2B), enabling extrapolation of each individual’s threshold (light intensity eliciting b-wave amplitude at 10% of maximum on the fitted Boltzmann curve) and slope (τ) for each ERG across sex and light categories. Used previously (Rosencrans et al., 2018), the Boltzmann equation was again deemed valid, as the mean (±SD) *r*^2^ of the fits for all individual scotopic and photopic curves were 0.95 (±0.06) and 0.97 (±0.03), respectively. The population-wide curves are shown in Figures 3A–D.

**FIGURE 4:**
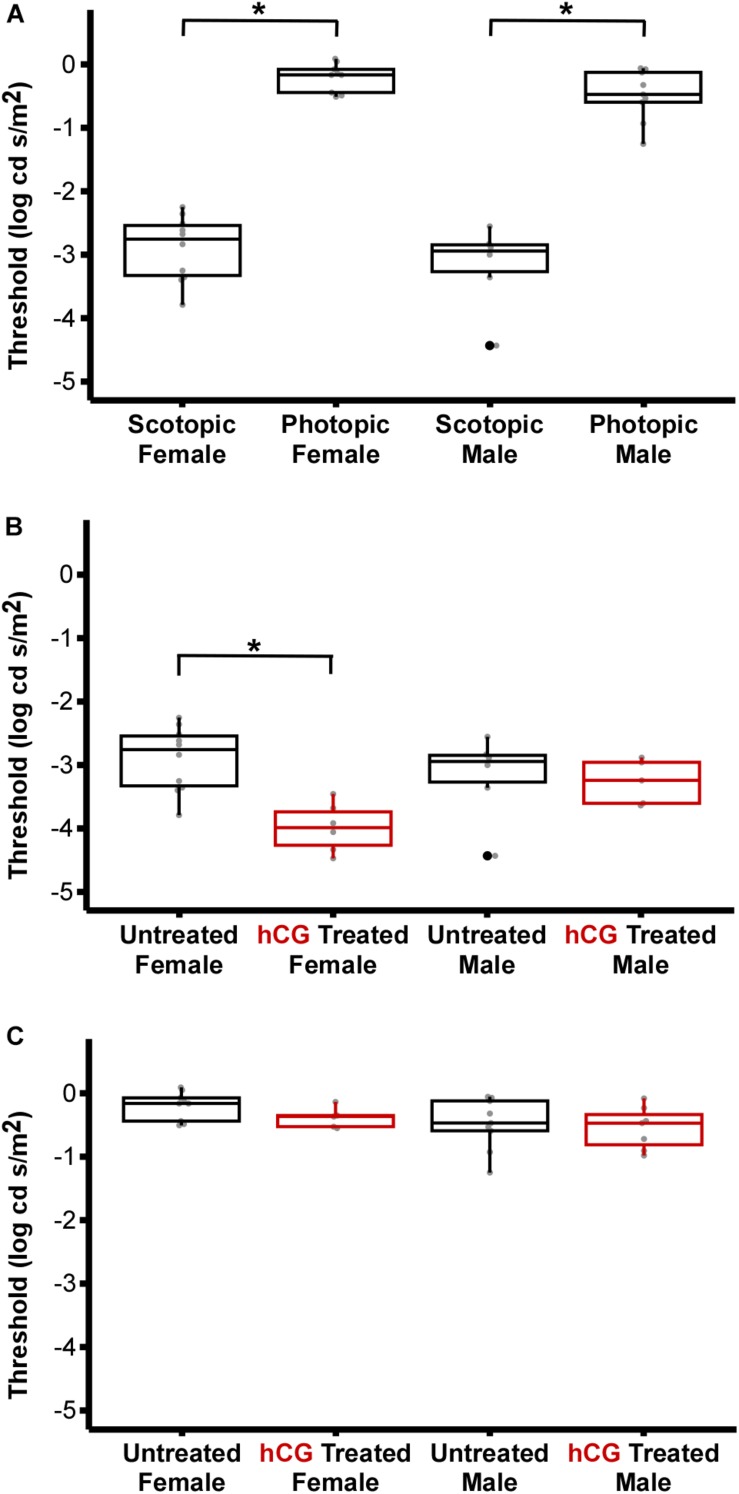
Comparison of ERG thresholds for males and females across lighting and hormone treatment conditions. **(A)** Within sex, scotopic vs. photopic thresholds. Scotopic are significantly lower than photopic (male, *P* < 0.0001; females, *P* < 0.0001). **(B)** Within sex, untreated vs. hCG treated scotopic thresholds. hCG lowered threshold in females (*P* < 0.0001), but not males (*P* = 1.000). **(C)** Within sex, untreated vs. hCG treated photopic thresholds. hCG had no effect in females (*P* = 0.9963) and males (*P* = 1.000). All numerical values and comparisons are in Tables 1, 2. For data dispersion presented here, the center line of each box indicates the median. The lower and upper hinges (i.e., the lower and upper boundaries of each box) mark the first and third quartiles, while each whisker stretches from the corresponding hinge to the furthest value no further than 1.5 times the interquartile range (distance between the first and third quartiles) from the hinge. Any data points beyond the whiskers are outlying points. Asterisks (^∗^) denote significant difference.

As expected, mean scotopic thresholds were significantly lower than those from photopic tests for both male and female túngara frogs, resulting from V-Log(I) curves shifting left on the *x*-axis (dimmer light) and indicating increased visual sensitivity under dark conditions. Mean (±SD) scotopic thresholds for males and females were −3.17 ± 0.67 and −2.90 ± 0.51 Log cd/m^2^, respectively. Photopic thresholds for males and females were −0.48 ± 0.40 and −0.20 ± 0.22 Log cd/m^2^, respectively (Figure 4A; see Tables 1, 2 for full results and statistical comparisons). Boltzmann curve slopes (τ) were significantly higher for scotopic than photopic curves for both sexes. Because the slope appears in the denominator of the exponent, higher slopes indicate a more gradual change in responses (larger dynamic range) under scotopic conditions (Tables 1, 2). Within each background lighting condition there were no significant differences between males and females for either threshold or Boltzmann slope (dynamic range) (Tables 1, 2). At the mechanistic level, the scotopic and photopic curves indicate rod and cone dominated response, respectively. Thus, for non-reproductive individuals, the latter result shows there is no evidence for sexual differences in retinal responses to white light within nocturnal and diurnal conditions.

### Effect of hCG on ERG Thresholds

Under scotopic conditions, female túngara frogs injected with hCG had increased visual sensitivity, as they exhibited V-Log(I) curves shifted to lower intensities (Figures 3E–H), resulting in a significantly lower mean threshold compared to untreated females, as well as untreated and hCG treated males (mean scotopic thresholds ± SD Log cd/m^2^: hCG females −4.22 ± 0.72; untreated females −2.90 ± 0.51; hCG males −3.26 ± 0.35; untreated males −3.17 ± 0.67). Males, in contrast, displayed no significant change in scotopic thresholds as a result of hCG treatment (Figure 4B and Tables 1, 2). Under photopic conditions, neither males nor females showed a change in visual sensitivity in response to hCG treatment (mean photopic thresholds ± SD Log cd/m^2^: hCG females −0.38 ± 0.17; untreated females −0.20 ± 0.22; hCG males −0.54 ± 0.34; untreated males −0.48 ± 0.40) (Figure 4C and Tables 1, 2). With regard to V-Log(I) slope, although a small difference appears between hCG treated male and female scotopic slopes, relative to individuals untreated with hCG, neither males nor females showed a change in Boltzmann slope in response to hCG treatment in either lighting condition (Tables 1, 2). This means dynamic range remained unchanged, as scotopic curves shifted, rather than stretched, in females to lower light responses. Thus, across the ERG measurements, the results indicate an effect of hormone treatment that is limited to lowering female threshold to light under scotopic (nocturnal) conditions only.

### Optical Anatomy and Sensitivity vs. ERG Threshold

**FIGURE 5:**
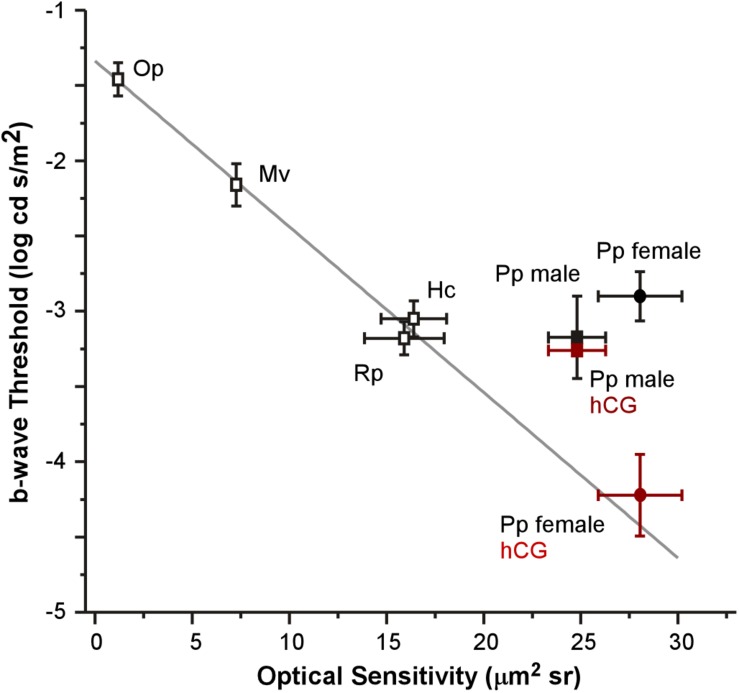
Relationship between mean (±SEM) optical sensitivities and ERG b-wave thresholds for five species: *Oophaga pumilio* (Op), *Mantella viridris* (Mv), *Hyla cinerea* (Hc), *Rana pipiens* (Rp), and *Physalaemus pustulosus* (Pp). Data (open symbols) and gray line fit for Op, Mv, Hc, and Rp are from Rosencrans et al. (2018). ERG thresholds for non-reproductive *P. pustulosus* (filled black symbols) are similar to those in other nocturnal species (non-reproductive), but do not match what is predicted by optical anatomy except for hCG treated females (red square: hCG males *P. pustulosus*; red circle: hCG female *P. pustulosus*).

Each anatomical component of the Land optical sensitivity equation was measured in untreated male and female túngara frogs in order to test how well the sensitivity predicted by their optical anatomy corresponded to the thresholds measured by their ERGs. Pupillary diameters (*A*: aperture) were determined using infrared photography, while focal lengths (*f*) were determined using flash-frozen ocular sections. Photoreceptor outer segment lengths and diameters were measured from high magnification micrographs. Mean (±SD) aperture (female 1.98 ± 0.09; male 1.98 ± 0.10 mm), focal length (female 1.57 ± 0.10; male 1.57 ± 0.07 mm), and outer segment diameters (female 7.13 ± 0.83; male 7.20 ± 1.08 μm) did not differ between males and females. Only photoreceptor length was dimorphic (female 69.40 ± 7.46; male 52.54 ± 6.65 μm) (Tables 1, 2 and Supplementary Figure S1), as females exhibited longer rod outer segments. Although this lengthening has the effect of increasing optical sensitivity (see Eq. 2), there was no significant optical sensitivity difference between males and females (female 28.05 ± 5.26; male 24.80 ± 3.56 μm^2^ sr) (Figure 5 *x*-axis, Tables 1, 2 and Supplementary Figure S1). The relationship between these optical sensitivities and ERG threshold did not approximate that predicted from other frog species, except for when thresholds were measured in hCG treated females (Figure 5; Rosencrans et al., 2018).

## Discussion

Our results implicate a hormonal modulation mechanism in the retina. The demonstrated effect was found in the b-wave of the ERGs, indicating that this modulation is likely occurring at or distal to the bipolar cells of the retina (Robson and Frishman, 1998). In particular, we found that hCG administration lowered retinal thresholds in female túngara frogs under nocturnal light conditions only (Figure 4). Such modulation is extremely relevant to the life history of these animals; during the breeding season, mate searching females select from male choruses at night (Ryan, 1985), and females use the visual cue of the vocal sac as an object of their searching behavior (Rosenthal et al., 2004; Taylor et al., 2008, 2011b; Taylor and Ryan, 2013). Thus, from a functional point of view, these heightened visual capabilities in a reproductive state are expected to benefit male detection under nocturnal conditions (Cummings et al., 2008). Although several traits in eye and retinal structure (e.g., optical parameters of the Land equation; increased rod number; rod receptor area pooling; temporal summation) are common in taxa under selection in constant low light conditions (Cronin et al., 2014), the benefit measured here is in the temporary context of mate choice. Along with other studies in túngara frogs and fish (Cummings et al., 2008; Butler et al., 2019), our data show that adaptations to low light may not be constant, but instead employed in a context dependent manner.

**FIGURE 6:**
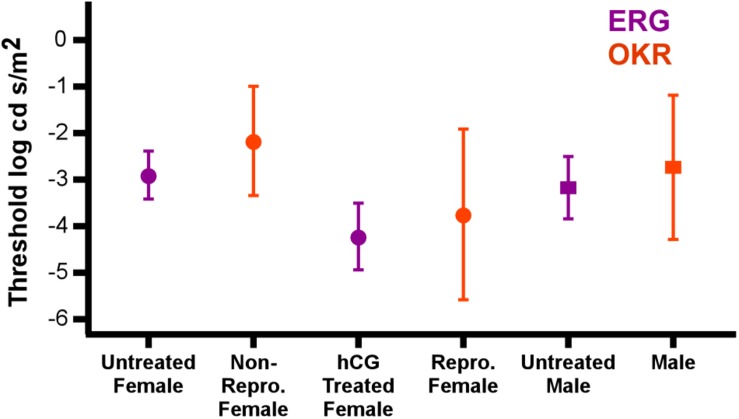
hCG induced modulation of retinal threshold in this study matches change in behavioral threshold in reproductive individuals. Points show comparison of the average (±SD) ERG thresholds with those for optokinetic responses (OKR) under scotopic conditions (Cummings et al., 2008). Data are separated by sex and reproductive state (hCG modulated for ERG; endogenous reproductive state for OKR). Circles and squares are females and males, respectively. ERG responses from this study are in purple, while optokinetic responses from Cummings et al. (2008) are in orange. Note that the stimulus illuminance in the behavioral tests (log Watts/cm^2^) was converted to log cd s/m^2^ for this comparison.

Confidence in our conclusion that hormonal modulation of visual behavior can be mediated in the retina is based on the close match in the ERG thresholds measured here (i.e., retinal change) and those independently found using optokinetic measurements in naturally reproductive and non-reproductive *P. pustulosus* (Cummings et al., 2008). Indeed, not only do our electrophysiological data reveal the same pattern found in their behavioral study, but also that the ERG thresholds were indistinguishable from the behavioral thresholds (see the data from the two separate studies combined in Figure 6). Albeit with the necessary addition of endocrine modulation, the data taken together provide another example of the close match between retinal and behavioral threshold in frogs (Aho et al., 1993). The similarity in thresholds across the studies also suggests the hormone injections resulted in an endocrine state matching that of natural reproductive behavior.

Under non-reproductive conditions, the relationship between túngara frog ERG thresholds and predicted sensitivity from optical anatomy deviates from the pattern established in previously described anuran species (Rosencrans et al., 2018). When compared to two nocturnal and two diurnal species, all of which were assumed to be non-reproductive (i.e., females were not gravid and did not deposit eggs on or after the day of the ERG), untreated túngara frogs exhibit higher thresholds than their optical anatomy would predict. Indeed, túngara ERG thresholds are similar to the other nocturnal species (∼-3.15 log cd s/m^2^) even though túngara optical anatomy predicts almost an order of magnitude lower threshold level. However, when treated with hCG, the lowered threshold exhibited by female túngara frogs more closely matches the pattern predicted by optical anatomy (Figure 5). This indicates that female túngara frogs are not operating at their maximum visual potential unless they are in a reproductive state, while males may not reach that potential at all. Without identification of the underlying modulatory mechanism, we can only speculate as to why low thresholds are temporary. At least one hypothesis for limited use of optical sensitivity is based on a need for diurnal vision. While highly sensitive scotopic vision should help females better locate a preferred male at night, it could lead to extensive diurnal visual saturation of rod vision that could be costly, such as through damage. However, admittedly this hypothesis is limited. First, based on V-Log(I) curves here, even at unmodulated thresholds, rod vision would likely already saturate during diurnal exposure. Second, while túngara frogs’ diurnal behavior is expected to include foraging (as their nights are often devoted to reproduction), currently little to nothing is known about what they do during the day and if they are exposed to light levels that would cause retinal damage in frogs (Fite et al., 1998). Of course, the underlying mechanism of the improved threshold, potentially including the modulation of retinal (phototransduction) noise (Aho et al., 1993; Pahlberg and Sampath, 2011) may be costly to maintain, as well. Nevertheless, our data support the hypothesis that there is a tradeoff to high optical sensitivity and that the suspected cost could be reduced by temporary hormonal modulation: females only have low thresholds when they are ready to mate and lose that sensitivity once the need for it has passed.

There are multiple hypotheses for why we do not see hCG modulation in males, despite their optical potential for heightened nocturnal sensitivity. Males may have less of an adaptive need for heightened nocturnal vision to perform their visually directed behavior. Although túngara frog courtship necessitates females approaching calling males, males may or may not approach a calling male and may only approach near a calling male, potentially to locate breeding sites (Ryan, 1985). Females may also need more complex visual information in their approach behavior, such as the need to locate a vocal sac inflating synchronously with the call that she prefers (Taylor and Ryan, 2013). From a mechanistic point of view, male and female vision could have different modulatory mechanisms, meaning our hCG protocol wasn’t sufficient to modulate vision in males. Indeed, whereas females are reproductive for only a limited amount of time (every 4–6 weeks) (Davidson and Hough, 1969) males call many nights throughout breeding season (Rand and Ryan, 1981). If reproductively related modulation for nocturnal vision occurred in males like in females, it would need to be repeatedly (every day) limited to a few hours of the circadian cycle. Evidence for such circadian changes in sensitivity has been shown in *Anolis* lizards (Shaw et al., 1993) and mediated by different hormonal mechanisms. Further testing is needed to determine if the lack of male modulation in our results is due to an insufficient protocol and/or reflective of evolutionary or biological constraints of males in the wild.

There are at least two important comparative implications of our dataset. The first is that there is potential for endocrine control of retinal processing of sexual signals across taxa. For example, the importance of visual cues in communication has been demonstrated in a variety of taxa, including humans (McGurk and MacDonald, 1976) and non-human animals, such as wolf spiders, horseshoe crabs, common garter snakes, and guppies (Reynolds et al., 1993; Hebets and Uetz, 2000; Shine and Mason, 2001; Taylor et al., 2005; Schwab and Brockmann, 2007). Even anuran amphibians (frogs and toads), which are typically nocturnal and have historically been the focus of bioacoustics studies, also use visual cues during communication (for review, see Hödl and Amezquita, 2001). Such signaling behavior includes foot-flagging in acoustically noisy environments (Grafe and Wanger, 2007); aposematic coloration (Maan and Cummings, 2009); and conspecific aggression elicited by vocal-sac pulsations (Narins et al., 2003, 2005) in the vibrantly pigmented dart-poison frog; and the effects of vocal sac movement in mate choice in the squirrel treefrog (Taylor et al., 2007). Given that most of these signals function in the context of mate choice, reproductive endocrine states could be modulating visual processing. The second comparative implication is that retinal sensitivity (and that of different sensory structures) may be more sexually dimorphic than previously thought, as such differences in sensitivity would only be revealed under particular endocrine states.

These two implications build upon a growing, yet limited, set of studies on reproductive hormonal modulation of receptor sensory function. For example, there are some notable studies demonstrating direct effects of hormones on the function of these structures. In plainfin midshipman, not only do testosterone and estrogen treatments of non-reproductive females increase the precision of temporal encoding in the inner ear of the male “hum” frequency, but estrogen receptor α (ERα) has been found in the inner ear as well (Sisneros et al., 2004). Additionally, females show increased auditory sensitivity at the inner ear during breeding season due to the reduced expression of the dopamine receptor D2a (Perelmuter et al., 2019). Zebra finches show sex differences in auditory brainstem responses, which implicates sex differences in inner ear responsiveness (Noirot et al., 2007), which could be due to differences in endocrine states, as both aromatase and ERα are present in hair cells of the basilar papillae of both males and females (Noirot et al., 2009). Such effects extend beyond the auditory system. Androgen treatment of juvenile male tinfoil barbs increased sensitivity of electro-olfactogram responses (olfactory epithelium) to prostaglandin (Cardwell et al., 1995), a common sex pheromone in fish (reviewed in Stacey and Sorensen, 1991; Stacey et al., 1994). In a weakly electric fish, *Apteronotus rostratus*, electroreceptor oscillation frequencies (a measure of electroreceptor tuning) decreased after estradiol implantation (Meyer et al., 1987), while weakly electric fish of the genus *Sternopygus* demonstrated lower electroreceptor oscillation frequencies in response to 5-α-dihydrotestosterone treatment (Keller et al., 1986).

Just as steroid hormones have been implicated in the modulation of other sensory systems, studies support a role in visual modulation. In humans, visual sensitivity is high during the time of ovulation and low during menstruation (Diamond et al., 1972), and estrogen receptor α was found in retinas of young women, but not of men or postmenopausal women (Ogueta et al., 1999). Studies have also demonstrated a neuroprotective effect of estrogen on the human retina and optic nerve (reviewed in Nuzzi et al., 2019). Aromatase (Gelinas and Callard, 1993) and estrogen receptor β (Tchoudakova et al., 1999) are both present in the retina of the goldfish, while a study of female western mosquitofish and sailfin mollies demonstrated various, species-specific effects of estrogen treatment on opsin and androgen receptor gene expression in the retina (Friesen et al., 2017). Androgen receptor β is present in eyes of both male and female three-spined sticklebacks (*Gasterosteus aculeatus*) (Hoffmann et al., 2012). However, despite these examples, commonly the literature on hormonal modulation of sensory systems focuses on central processing and not the receptor organs, including in frogs (Yang et al., 2007; Chakraborty and Burmeister, 2015). When taken together with our data, these examples from the literature implicate a potentially wide expression of sensory organ modulation with large capacity for sensitivity change. Here, our data shift threshold by 1.32 logarithmic units (Log cd s/m^2^), a > 20-fold change in threshold.

Putting the modulatory and anatomical differences found in our data into a broader comparative context is limited by the fact that sexual dimorphism in eyes seems to be largely understudied, especially amongst vertebrates. Examples across taxa include houseflies, *Musca domestica*, which have a region of their eyes with larger ommatidial facet lenses (Beersma et al., 1975; Land and Eckert, 1985), called the ‘love spot,’ that is unique to males. Photoreceptors in this region can code higher velocities and smaller targets than can female photoreceptors (Hornstein et al., 2000), which may explain sex differences in tracking behavior (Wehrhahn, 1979). In the butterfly *Heliconius erato*, females express two ultraviolet opsin proteins, while males only express one, which may be due to the female’s need to discriminate conspecifics from heterospecifics (McCulloch et al., 2016). With respect to a vertebrate, a more recent example of sexual dimorphism that is quite relevant to our study is modulation of the visual system by hormones in the mouthbrooding African cichlid, *Astatotilapia burtoni*, where ovulated females have a higher expression of sex steroids in retinal tissue as well as heightened retinal sensitivity to wavelengths of light that are reflective of male coloration (Butler et al., 2019). Although our data in this study do reveal a significant difference in one cellular dimension, outer segment length, to our knowledge it is still unknown if such a difference exists in other taxa.

There are several limitations to this study. The electroretinogram technique, while appropriate to demonstrate a change in the stimulus-response properties in the retina, cannot determine the particular underlying modulatory mechanism and whether other modulator sites exist (e.g., more central neural mechanisms). hCG binds to luteinizing hormone receptors, which stimulates the gonads of both males and females to release steroid hormones into the bloodstream (Ascoli et al., 2002; Menon and Menon, 2012). Thus, hCG itself may not be acting on retinal targets, as previous studies have shown that estradiol alone similarly induces the reproductive state in these frogs. Furthermore, the modulation of reproductive behavior and hormonal titer are reduced when hCG is combined with fadrazole to block estradiol production (Chakraborty and Burmeister, 2009). Additional studies are needed to identify the hormonal receptors (i.e., their cellular and/or subcellular modulatory targets) present in the túngara frog retina and how hormonal signals change the stimulus-induced response. Our data on modulation of the b-wave points toward modulatory targets at least at the level of the inner nuclear layer.

## Conclusion

We have shown that inducing a reproductive state in túngara frogs via hCG injection significantly increases the females’ scotopic retinal sensitivity. This implicates the retina in the mechanistic explanation of previously found hormonal effects on visual behaviors (OKR) in this species and demonstrates that endocrine regulation of receptor organs can have drastic effects on behavior. Such a finding in the receptor organ is relatively novel, as studies of hormonal modulation of behaviors have historically focused on higher-level sensory processing and decision-making in the central nervous system. We do not believe that túngara frogs are unique in this regard; the prevalence of both vision and hormonal modulation in reproductive behaviors make it very likely that hormones could be influencing behaviors by way of the visual system in many animals. More studies in a variety of taxa are necessary to determine how prevalent and varied this modulatory mechanism is in nature.

## Data Availability Statement

The datasets generated for this study are available on request to the corresponding author.

## Ethics Statement

The animal study was reviewed and approved by the Institutional Animal Care and Use Committees of The University of Texas at Austin, Louisiana State University Health Sciences Center, New Orleans, and the Smithsonian Tropical Research Institute.

## Author Contributions

CL and HF conceived and designed the study, and analyzed the data. CL, HF, and MR wrote the manuscript. CL, RR, and WW conducted the study and collected the data. RR, HF, and WG developed the protocols for ERG recordings and retinal morphology measurements. MR and NB provided funding and all laboratory resources.

## Conflict of Interest

The authors declare that the research was conducted in the absence of any commercial or financial relationships that could be construed as a potential conflict of interest.
