# Supplementary figures and images for: Reproductive State Modulates Retinal Sensitivity to Light in Female Túngara Frogs
Source: Front Behav Neurosci. 2020 Jan 21;13:293. doi: 10.3389/fnbeh.2019.00293 (PMC6985269; doi:10.3389/fnbeh.2019.00293)

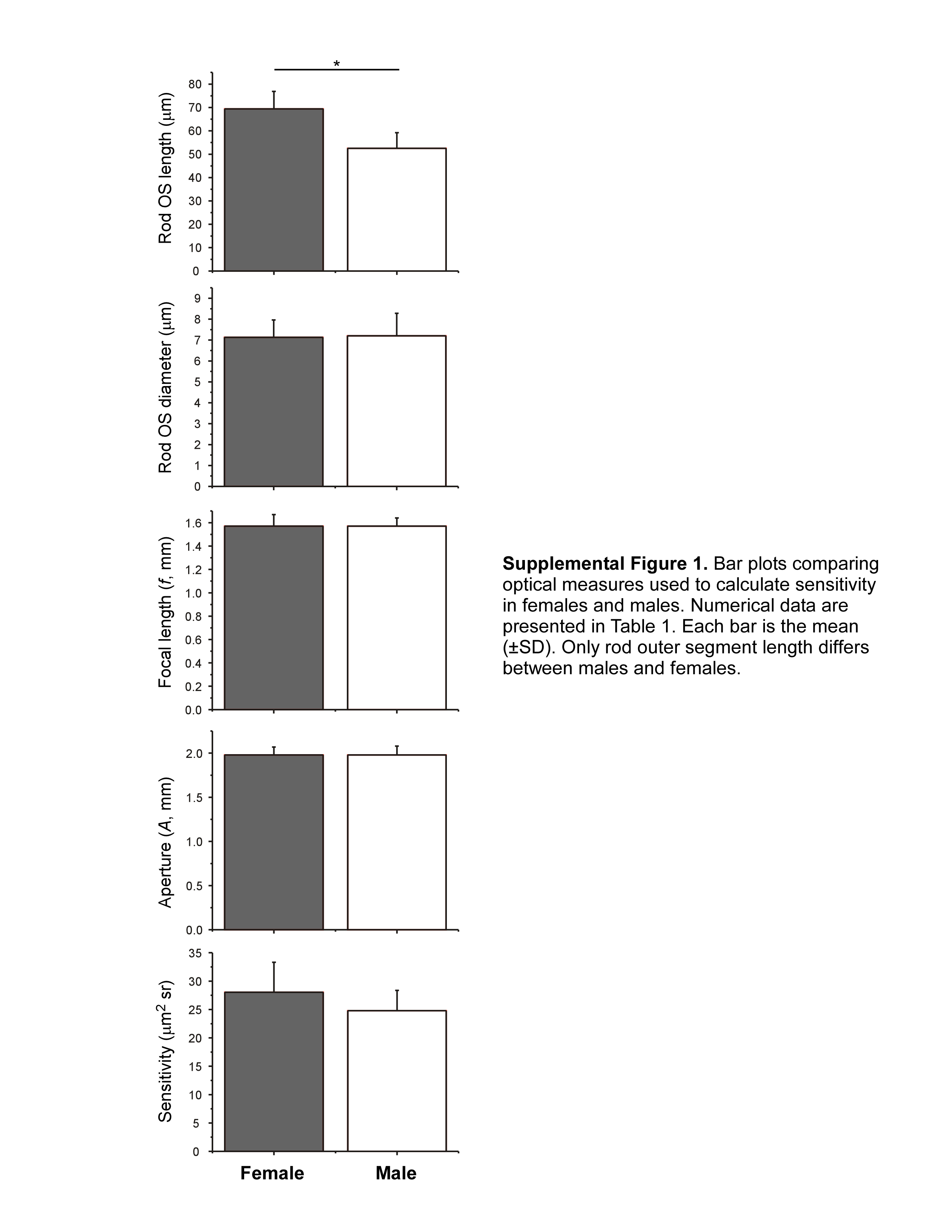

Supplement: Supplementary file 1 [file Image_1.TIF]
